# Characterization of VAMP2 in *Schistosoma japonicum* and the Evaluation of Protective Efficacy Induced by Recombinant SjVAMP2 in Mice

**DOI:** 10.1371/journal.pone.0144584

**Published:** 2015-12-07

**Authors:** Qian Han, Yang Hong, Zhiqiang Fu, Min Zhang, Xiaodan Cao, Yantao Liu, Shuai Ma, Yuntao Guo, Ke Lu, Chuangang Zhu, Jiaojiao Lin

**Affiliations:** 1 National Laboratory of Animal Schistosomiasis Control/Key Laboratory of Animal Parasitology, Ministry of Agriculture, Shanghai Veterinary Research Institute, Chinese Academy of Agricultural Sciences, Shanghai, People’s Republic of China; 2 College of Animal Science and Technology, Henan University of Science and Technology, Luoyang, People’s Republic of China; 3 Institute of Animal Sciences, Chinese Academy of Agricultural Sciences, Beijing, People’s Republic of China; 4 Jiangsu Co-innovation Center for Prevention and Control of Important Animal Infectious Diseases and Zoonoses, Yangzhou, China; George Washington University School of Medicine and Health Sciences, UNITED STATES

## Abstract

**Background:**

The outer-tegument membrane covering the schistosome is believed to maintain via the fusion of membranous vesicles. Fusion of biological membranes is a fundamental process in all eukaryotic cells driven by formation of trans-SNARE (soluble N-ethylmaleimide-sensitive factor attachment protein receptor) complexes through pairing of vesicle associated v-SNAREs (VAMP) with complementary t-SNAREs on target membranes. The purpose of this study was to characterize *Schistosoma japonicum* vesicle-associated membrane protein 2 (SjVAMP2) and to investigate its potential as a candidate vaccine against schistosomiasis.

**Methodology/Principal Findings:**

The sequence of SjVAMP2 was analyzed, cloned, expressed and characterized. SjVAMP2 is a member of the synaptobrevin superfamily harboring the v-SNARE coiled-coil homology domain. RT–PCR analysis revealed that significantly higher SjVAMP2 levels were observed in 14-, 28- and 42-day-old worms, and SjVAMP2 expression was much higher in 42-day-old female worms than in those male worms. Additionally, the expression of SjVAMP2 was associated with membrane recovery in PZQ-treated worms. Immunostaining assay showed that SjVAMP2 was mainly distributed in the sub-tegument of the worms. Western blotting revealed that rSjVAMP2 showed strong immunogenicity. Purified rSjVAMP2 emulsified with ISA206 adjuvant induced 41.5% and 27.3% reductions in worm burden, and 36.8% and 23.3% reductions in hepatic eggs in two independent trials. Besides, significantly higher rSjVAMP2-specific IgG, IgG1, IgG2a levels were detected in rSjVAMP2-vaccinated mice.

**Conclusion:**

Our study indicated that SjVAMP2 is a potential vaccine candidate against *S*. *japonicum* and provided the basis for further investigations into the biological function of SjVAMP2.

## Introduction

Schistosomiasis is an important parasitic disease epidemic in the tropics and subtropics that infects more than 200 million people in over 70 countries, causes an estimated 280,000 deaths annually, and endangers an additional 650 million people worldwide [1,2]. Moreover, schistosomiasis represents an increasing problem in non-endemic areas due to environmental change and the growing number of immigrants and tourists [3,4]. *Schistosoma japonicum* (*S*. *japonicum*) is the causative agent of schistosomiasis in China. Despite decades of intense efforts to control *S*. *japonica*, schistosomiasis is still a major public health problem in China, where it is endemic in seven provinces in which an estimated 286,836 cases were reported in 2011. And reinfection remains a major challenge to the control of the disease [5]. Therefore, the development of an effective molecule as a potential vaccine to protect both humans and domestic animals is an important goal.

Schistosome is a genus of complex multicellular pathogen that have co-evolved with their mammalian hosts despite the development of a pronounced immune response [6]. The tegument exposed to the host immune systems directly is of crucial importance for the schistosome to survive for decades within the inhospitable environment [7,8]. In addition, the tegument is a dynamic organ involving in nutritional uptake, structural support, signal transduction and immune evasion [9,10]. And the dual membrane–membranocalyx complex covering the surface layer of the schistosome tegument is dynamically replaced by a burst outward of membranous vacuoles that are synthesised in the cytons, and then passed to the tegument [9,11]. Therefore, components of the tegument, surface membrane-associated and surface-exposed proteins are considered as perfect source for antigens in the development of vaccines and diagnosis tests [12,13]. A proteomics study of *S*. *japonicum* in our laboratory showed that vesicle-associated membrane protein 2 (VAMP2) is a tegument surface protein. Besides, VAMP2 is a key part of the soluble N-ethylmaleimide-sensitive factor attachment protein receptor (SNARE) that is required for membrane fusion.

With few exceptions, the fusion of biological membranes, a fundamental process governing the transport of cargo molecules, such as the trafficked proteins, hormones, and neurotransmitters throughout the secretory and endocytic pathways, is driven by the formation of trans-SNARE complexes [14,15]. SNAREs are compartment-specific proteins that consist of VAMP2 bound to the synaptic vesicle (v-SNARE), syntaxin and SNAP25 on the target membrane (t-SNARE). By pairing v-SNAREs with cognate t-SNAREs, a bundle of four helices (SNAREpins), three derived from the t-SNARE and the fourth from the cognate v-SNARE, is assembled, bringing the two lipid bilayers into close proximity, finally culminating in membrane fusion [16,17]. SNAREpin assembly is a sequential, two-step folding pathway, and each step has specific and distinct functions. A previous study has shown that the N-terminal domain (NTD) of the v-SNARE docking to the t-SNARE is the rate-limiting step, which suggests that VAMP2 plays a crucial role in membrane fusion, mediating protein trafficking and the secretion of physiological mediators [18,19].

The role of VAMP2 is of vital importance in other processes in addition to membrane fusion and vesicular transport. In a previous study, Yuki *et al*. have suggested that VAMP2 engages in the trafficking of recycling endosomes, and it also can be used as a molecular marker for both quiescent satellite cells and myotubes, but not for proliferating myoblasts [20]. Lin *et al*. have reported that thyroid hormone may promote glucose uptake via enhancing insulin-induced phosphorylation of Akt and subsequent translocations of VAMP2 and GLUT4 in 3T3-L1 adipocytes [21]. Additionally, by reducing VAMP2 expression in a murine model of epilepsy, mice showed significant reductions in potassium-evoked glutamate release, which led to a kindling-resistant phenotype [22]. Undoubtedly, VAMP2 is a key molecule in many processes, but how it mediates the development of *S*. *japonicum* is still unknown.

In the present study, we described the cloning, expression, and characterization of SjVAMP2, including the distribution of the protein in *S*. *japonicum*, its transcript levels in worms of different developmental stages, as well as worms treated with the anti-schistosome drug praziquantel (PZQ). The protective efficacy induced by recombinant SjVAMP2 (rSjVAMP2) in mice was also evaluated.

## Materials and Methods

### Ethics Statement

All animal experiments were approved by the Animal Care and Use Committee of Shanghai Veterinary Research Institute, Chinese Academy of Agricultural Sciences for the use of laboratory animals (Permit ID Number: SHVRI 2013–0909). All animals were housed in a 12 h light/dark cycle and virus/antigen-free facility with controlled temperature and humidity, and were provided with water and food ad libitum, and were monitored daily. For euthanasia, animals were deeply anesthetized with CO_2_ followed by cervical dislocation. We made all efforts to minimize animal suffering and discomfort in accordance with the guidelines of the Regulations for the Administration of Affairs Concerning Experimental Animals, China.

### Parasites and animals

Specific pathogen free (SPF) male BALB/c mice aged 6 weeks were purchased from the Shanghai Laboratory Animal Center, CAAS (Shanghai, China). Seven-, 14-, 21-, 28-, 35-, and 42-day-old schistosomes were collected from New Zealand rabbits percutaneously infected with 1000 to 6000 cercariae of S. j*aponicum* that were maintained and released from Oncomelania hupensis snails infected with a Chinese mainland strain of *S*. *japonicum*, which was supplied by the Shanghai Veterinary Research Institute, CAAS.

### Bioinformatic analysis

Initially, the sequence of SjVAMP2 was retrieved on the UniProt website (http://www.uniprot.org/uniprot/). This was followed by BLASTp analysis to confirm annotation features and to identify homologous sequences from different organisms in the NCBI database. These sequences were aligned using the multiple sequence alignment program ClustalW 2.0 (http://www.ebi.ac.uk/Tools/clustalw2/index.html), and conserved protein domains or motifs were predicted using MEME (v4.1) (http://meme.nbcr.net/meme/tools/meme). The tertiary structure and transmembrane helix of SjVAMP2 were predicted using Phyre2 [23]. Additionally, phylogenetic analyses were performed using MEGA 5.1 software (http://www.megasoftware.net/) by constructing a consensus tree of the VAMP2 sequences from various organisms to infer the evolutionary history of the taxa.

### Gene cloning and plasmid preparation

Based on the ORF of SjVAMP2 (GenBank accession: AAP05935.1), the sequence was amplified with primers 5'-GTGGAATTCATGTCAGCCGACAC-3' (*EcoRI* restriction site underlined) as the sense primer and 5'-GTTCTCGAGTCACTGAGTAGCACTTCCA-3' (*XhoI* restriction site underlined) as the antisense primer. PCR amplification was conducted under the following conditions: 1 min hold at 94°C, 30 cycles of 94°C for 30 s, 56.5°C for 1 min, and 72°C for 1min, followed by a hold for 10 min at 72°C to ensure full extension. The PCR product was purified, and ligated to the pMD19-T vector (Takara) at 16°C overnight, and transformed into *Escherichia coli* DH5α cells (Tiangen). Clones were selected, screened, and subjected to DNA sequence analysis.

### Real-time PCR analysis

Total RNA of parasites at different developmental stages, including cercariae, 7-, 14-, 21-day-old schistosomula, 28-, 35-, 42-day-old adult worms, eggs, as well as 42-day-old males and females were transcribed using PrimeScript^TM^ RT reagent Kit (TaKaRa). Considerable care was taken to ensure that all the total RNA samples used were of high quality (A260/A280 ≥1.7 in nuclease-free water) with minimal degradation, as recommended by Bustin and Nolan (2004). Samples were treated with RNase-free DNaseI before complementary DNA (cDNA) was synthesized from total RNA using the RNeasy Mini Kit (Qiagen). Then, cDNA samples adjusted to 5 ng were used as templates for real-time PCR. PCR amplification was performed with SYBR Premix Ex Taq^TM^ kit (TaKaRa) in an ABI PRISM 7500 Fast Real-Time PCR System instrument. SjNADH was used as an endogenous control [24]. Negative controls without templates were conducted at the same time. Melting curves were used to optimize the cycling conditions and to verify the specificity of the PCRs. All RT–PCR experiments were performed in triplicate, and the confidence threshold (CT) value was analyzed with ABI PRISM 7500 software. The SjVAMP2 RT-PCR primer pairs were 5'-ACAACCTCGACCACAGAACAAG-3'(sense) and 5'-TTCCTGCACTAGCCTCGAATTG-3'(antisense), and the primers of SjNADH were 5'-CGAGGACCTAACAGCAGAGG-3' (sense) and 5’-TCCGAACGAACTTTGAATCC -3' (antisense).

RT–PCR was also performed to detect SjVAMP2 expression changes while the worms were exposed to incomplete treatment or treatment doses of PZQ. Seventy-two mice divided randomly into PZQ-treated and untreated groups of six mice each were challenged with cercariae. Thirty-five days after challenge, mice in the treated groups were treated with an incomplete treatment dose of PZQ at 40 mg/kg, or a treatment dose of 200 mg/kg in carboxymethyl cellulose (CMCNa), while the control groups were treated with CMCNa only. After drug administration, both treated and untreated mice were sacrificed at 30 min, 4 h, 12 h, and 36 h, respectively. The effect of PZQ on SjVAMP2 transcription was analyzed by RT–PCR. All reactions were carried out in three biological replicates.

### Expression and purification of rSjVAMP2 protein

The purified recombinant plasmid, pMD19-T-SjVAMP2 was digested with *EcoRI* and *XhoI*, and then was ligated to the pET28a (+) vector (Invitrogen) overnight at 16°C using T4 DNA ligase (TaKaRa). The resulting plasmid, pET28a(+)/SjVAMP2, in which a His tag was fused to SjVAMP2, was transformed into *Escherichia coli* BL21(DE3) cells (Tiangen). Transformed bacterial cells were induced by isopropyl-1-thio-b-D-galactoside (IPTG) to a final concentration of 1 mM at 20°C for 6h, then pelleted by centrifugation at 12,000 *g* for 15 min, resuspended in phosphate-buffered saline (PBS, pH 7.4), and frozen at −80°C until used. Frozen cells were lysed by sonication and pelleted by centrifugation at 4°C, 12,000 *g* for 15 min. The insoluble pellet was resuspended in binding buffer containing 8 M urea, while the soluble supernatant was conserved. The crude extract was analyzed by SDS–PAGE.

The supernatant containing rSjVAMP2-His was purified with an Ni-NTA His-Bind Resin (Qiagen GmbH, Hilden, Germany) and dialyzed against PBS (pH 7.4), containing decreasing concentrations of urea (6, 4, 3, 2, and 1 M) and then PBS only. rSjVAMP2-His was concentrated using Centricon microconcentrators (Amicon Millipore, Shanghai, China), and the protein concentration was determined by the Bradford method using bovine serum albumen (BSA) as a protein standard.

### Preparation of mouse antibody specific to rSjVAMP2

Six BALB/c mice were immunized subcutaneously with three doses of purified rSjVAMP2 at 2 week intervals (20 μg at each injection). Immune serum specific to rSjVAMP2 was harvested 1 week after the last injection and stored at −20°C for further use.

### Western Blotting Analysis

Native antigens, SWAP (soluble adult worms antigen preparation) and SEA (soluble egg antigen) made in our lab were subjected to 12% SDS–PAGE and then electroblotted onto a nitrocellulose membrane (Whatman, German). Non-specific protein binding was blocked by incubating the membrane in PBS containing 0.05% Tween 20 (PBST) and 5% skim milk at 4°C overnight. After washing with PBST, the membrane was incubated with mouse anti-rSjVAMP2 serum (1:100 dilution) for 1 h at 37°C with constant shaking. Following three 5-min washes with PBST, horseradish peroxidase (HRP)-conjugated goat anti-mouse IgG at a dilution of 1:2000 was used as a secondary antibody, and the signal was subsequently detected with the DAB Substrate Solution (Tiangen Biotech, Beijing, China). Serum from non-immunized mice was used as a negative control.

### Immunolocalization of SjVAMP2

Fresh collected adult worms (28 days) were fixed in 4% paraformaldehyde immediately, then dehydrated in an ascending ethanol series, cleared with xylene, embedded in paraffin wax, and finally cut into 5 μm sections for immunostaining assays. The sections were first deparaffinized, hydrated, and quenched with 3% H_2_O_2_ at room temperature for 20 min to remove the endogenous peroxidase, then boiled in citric acid/sodium citrate buffer solution(0.01M, pH6.0) for 20 min to expose the antigens sufficiently and cooled down naturally. After the sections were incubated with 10% goat serum at 4°C overnight, the samples were probed with rSjVAMP2-immunized mouse serum diluted 1:8000 for 2 h at 37°C, along with the negative control group incubated with serum from non-immunized mice. After three 5-min washes in PBST after each incubation step, the second antibody and the third antibody in the SP (Streptavidin-Peroxidase) Kit (ZSGB-BIO, China, Beijing) and DAB (DAKO, Denmark) were used according to user manual recommendation. Sections were then washed in PBST, counterstained with hematoxylin, mounted in neutral balsam, and photographed using a Nikon E80i microscope.

### Mouse vaccination experiment

Six-week-old male BALB/c mice were allocated randomly into three groups of ten mice each. Montanide ISA206 was used as the vaccine adjuvant since it can induce short- and long-term protective immune responses. The vaccination group was immunized subcutaneously three times with 100 μl of rSjVAMP2 (20 μg/mouse) in ISA206 adjuvant at 2-week intervals (on weeks 0, 2 and 4), while the two control groups were injected with ISA206 adjuvant in PBS or PBS only, respectively. Two weeks after the third immunization, all mice were challenged percutaneously with 40 ± 2 cercariae. All mice were sacrificed 6 weeks post-challenge by portal perfusion. Worms in the perfusate sediment were collected and counted, and the intestinal mesenteric vessels of each mouse were examined for residual worms. Each hepatic lobe was homogenized in PBS and digested in 10% NaOH for 15 min at 56°C, and then the eggs in three 100 μl subsamples of the digestion fluid of each sample were counted. The mean count was taken as the number of eggs in each sample tested, and was converted to eggs per gram (EPG) in the liver. A repeat trial was performed under the same condition 5 months later. Vaccine-induced protection was measured by the percentage of worm or hepatic egg reduction in the vaccinated groups using the following formula:

Worm/egg reduction rate (%) = (numbers of worms/eggs in the ISA206 adjuvant group − numbers of worms/eggs in the rSjVAMP2 group)/numbers of worms/eggs in the ISA206 adjuvant group × 100%.

### Detection of specific antibody against rSjVAMP2

Samples of blood were collected from each mouse by retro-orbital bleeding before vaccination, 1 week after each vaccination, and before sacrifice. ELISA assay was performed to detect specific IgG, IgG1, and IgG2a antibodies against rSjVAMP2. Protocol optimization and dilution of testing reagents was determined by checkerboard titration analysis. Each testing well of 96-well microtiter Costar plates (Corning Life Science, Tewksbury, MA, USA) was coated with 100 μl of rSjVAMP2 (10 μg/ml) diluted in carbonate bicarbonate buffer (pH 9.6) at 4°C overnight, and washed three times with PBST and blocked in PBST-5% skimmed milk at 37°C for 1 h. Then, the wells were washed three times and incubated with the tested sera samples (1:100 dilutions) for 2 h at 37°C. After two additional washes, goat anti-mouse IgG-conjugated HRP (Sigma–Aldrich) (1:3000 dilution) was added and incubated at 37°C for 1 h. Afterwards, washes were performed and tetramethyl benzidine dihydrochloride (TMB, Sigma–Aldrich) substrate was added and incubated at 37°C in the dark for 10 min. Finally, the reaction was stopped with 2 M sulfuric acid, and the absorbance was read at 450 nm on a microplate reader (BioTek, Winooski, VT, USA). All samples were run in triplicate, and negative controls with naive mice serum were performed at the same time to ensure that the environment was not contaminated. To detect IgG1 and IgG2a, goat anti-mouse IgG1- or IgG2a-conjugated HRP (Sigma–Aldrich) (1:2000 dilutions) were used as secondary antibodies as described for IgG detection.

### Statistical analysis

All data acquired in this study were generated from at least three replicates of independent experiments using identical protocols. One-way analysis of variance (ANOVA) or T-tests were performed using SPSS for Windows 13.0. The data were expressed as means ± SEM; P values < 0.05 and P values < 0.001 were considered significant and highly significant, respectively.

## Results

### Bioinformatic analysis of SjVAMP2

**Fig 1:**
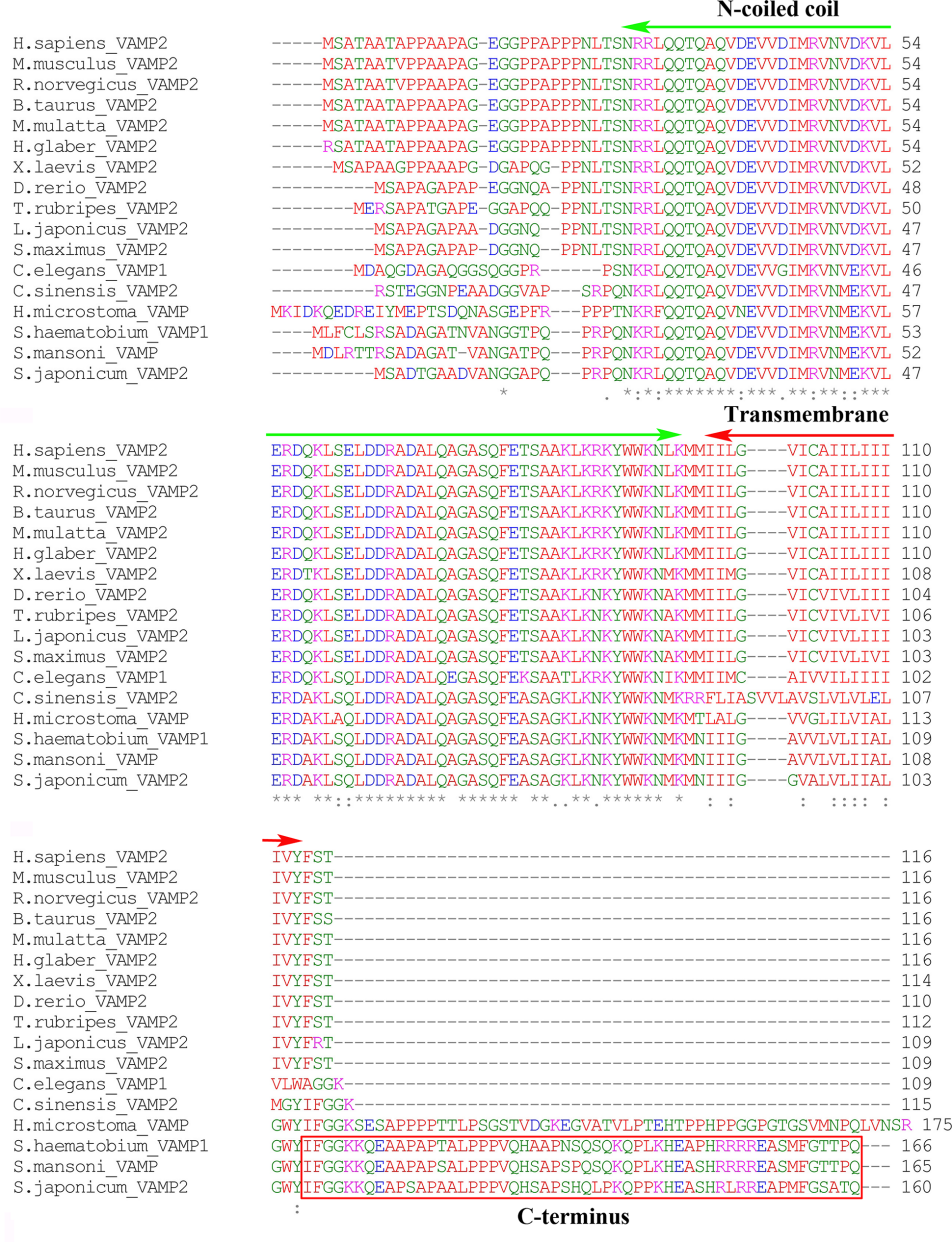
Multiple alignment of SjVAMP2 with the VAMP2 of other animals. The color of letters denotes physicochemical properties of amino acids: red—small and hydrophobic; green—hydroxyl, sulfhydryl, amine and G; magenta—basic; and blue—acidic. The conserved domain of amino acid residues is displayed with different consensus symbols: An asterisk (*) indicates positions that have a single, fully conserved residue; a colon (:) indicates conservation between groups of strongly similar properties–scoring > 0.5 in the Gonnet PAM 250 matrix; a period (.) indicates conservation between groups of weekly similar properties–scoring ≤ 0.5 in the Gonnet PAM 250 matrix. N-coiled coil homology and transmembrane domains are showed in regions of bars with green or red colors respectively. Red boxed residues are C-terminus conserved domain in VAMP of schistosome. Aligned sequences are *Homo sapiens* (NP_ 055047.2), *Mus musculus* (NP_ 033523.1), *Rattus norvegicus* (NP_036795.1), *Bos taurus* (NP_776908.1), *Macaca mulatta* (EHH24500.1), *Heterocephalus glaber* (EHB04514.1), *Xenopus laevis* (NP_001080944.1), *Danio rerio* (NP_956299.1), *Takifugu rubripes* (NP_003976339.1), *Lateolabrax japonicus* (AAT67161.2), *Scophthalmus maximus* (CCD74823.1), *Caenorhabditis elegans* (NP_504688.1), *Clonorchis sinensis* (GAA52723.1), *Hymenolepis microstoma* (CDS31115.1), *Schistosoma haematobium* (KGB32859.1), *Schistosoma mansoni* (CCD74823.1), and *Schistosoma japonicum* (AAP05935.1).

**Fig 2:**
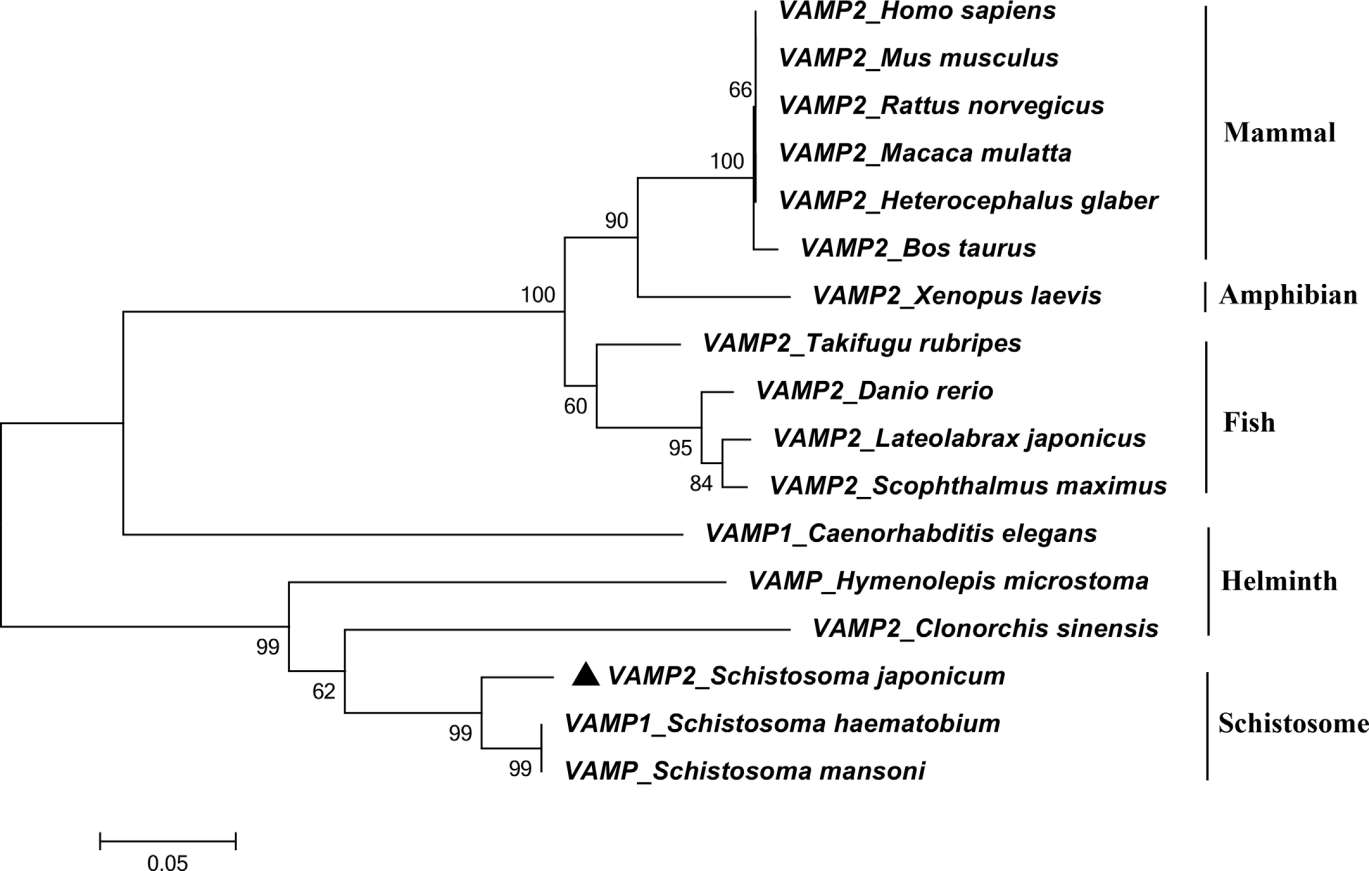
A phylogenetic tree of SjVAMP2 with those of other animals. The neighbor-joining tree was constructed based on VAMP2 amino acid sequences that were retrieved from GenBank. The number at each node shows the bootstrap value (1000 replicates). (NP_055047.2, NP_033523.1, NP_036795.1, NP_776908.1, EHH24500.1, EHB04514.1, NP_001080944.1, NP_956299.1, NP_003976339.1, AAT67161.2, CCD74823.1, NP_504688.1, GAA52723.1, CDS31115.1, KGB32859.1, CCD74823.1, AAP05935.1) is GenBank accession number.

BLASTp and MEME analysis of VAMP2 of different organisms revealed that SjVAMP2 appeared to be a member of the synaptobrevin superfamily harboring the v-SNARE coiled-coil homology domain as shown in Fig 1. In the transmembrane domain, residues were hydrophobic. Several other key residues were also conserved. Notably, VAMP sequences from *Schistosome* (*S*. *japonicum*, *S*. *mansoni*, and *S*. *haematobium*) contained a conserved C-terminus, not exist in other homologous sequence (Fig 1). The conserved C-terminus domain of SjVAMP2 was in the cytoplasm, N-coiled coil homology domain was extracellular, and 87 to 109 amino acid residues was located at the transmembrane domain (S1 Fig). A conceptual tertiary structure of this polypeptide showed that N-coiled coil domain had 2 ɑ-helix, transmembrane domain had 1 TM helix (S2 Fig). A phylogenetic analysis showed that SjVAMP2 was grouped with homologs of schistosome such as *Schistosoma mansoni* and *Schistosoma haematobium*. The mammalian VAMP2 formed a major clade (Fig 2).

### mRNA expression level of SjVAMP2 in different developmental stages of *S*. *japonicum*

**Fig 3:**
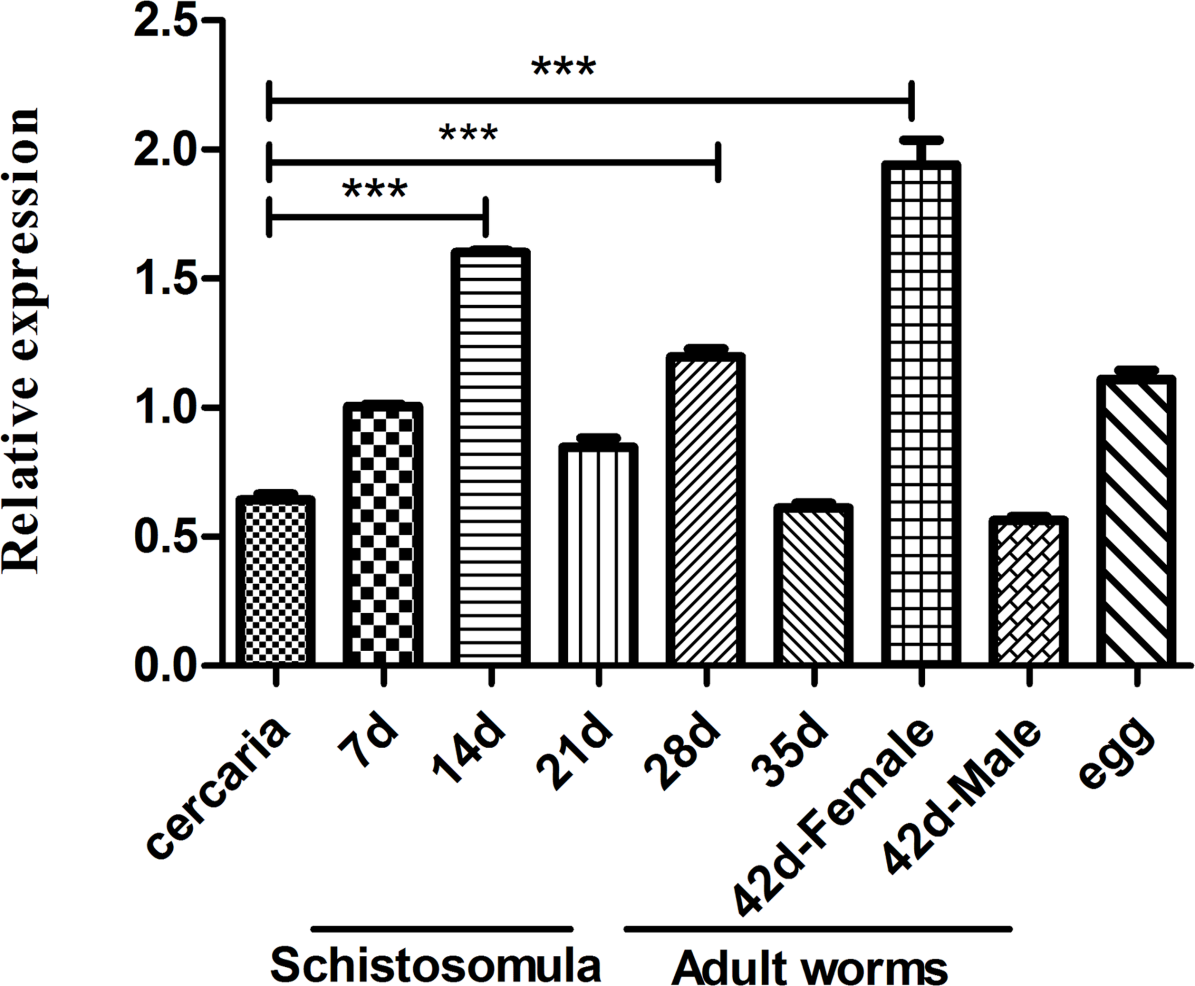
Relative transcriptional level of SjVAMP2 mRNA in different developmental stages. SjVAMP2 mRNA expression in different stages of *S*. *japonicum*, including cercariae, schistosomula, adult worms, eggs, as well as male and female adult worms, was analyzed by RT-PCR. Data were normalized against amplification of an internal housekeeping control gene, NADH, for three independent experiments.

Different developmental stages, including cercariae, schistosomula, adult worms and eggs, as well as 42-day-old males and females, were analyzed by real-time PCR, and SjVAMP2 mRNA expression exhibited different in all developmental stages tested. Notably, significantly higher expression levels of SjVAMP2 were found in 14-, 28-, and 42-day-old worms compared to carecia (p<0.001). Moreover, a substantially higher expression level was exhibited in 42-day-old female worms about 3-fold than in male worms (Fig 3).

### Effect of PZQ on SjVAMP2 Transcription

**Fig 4:**
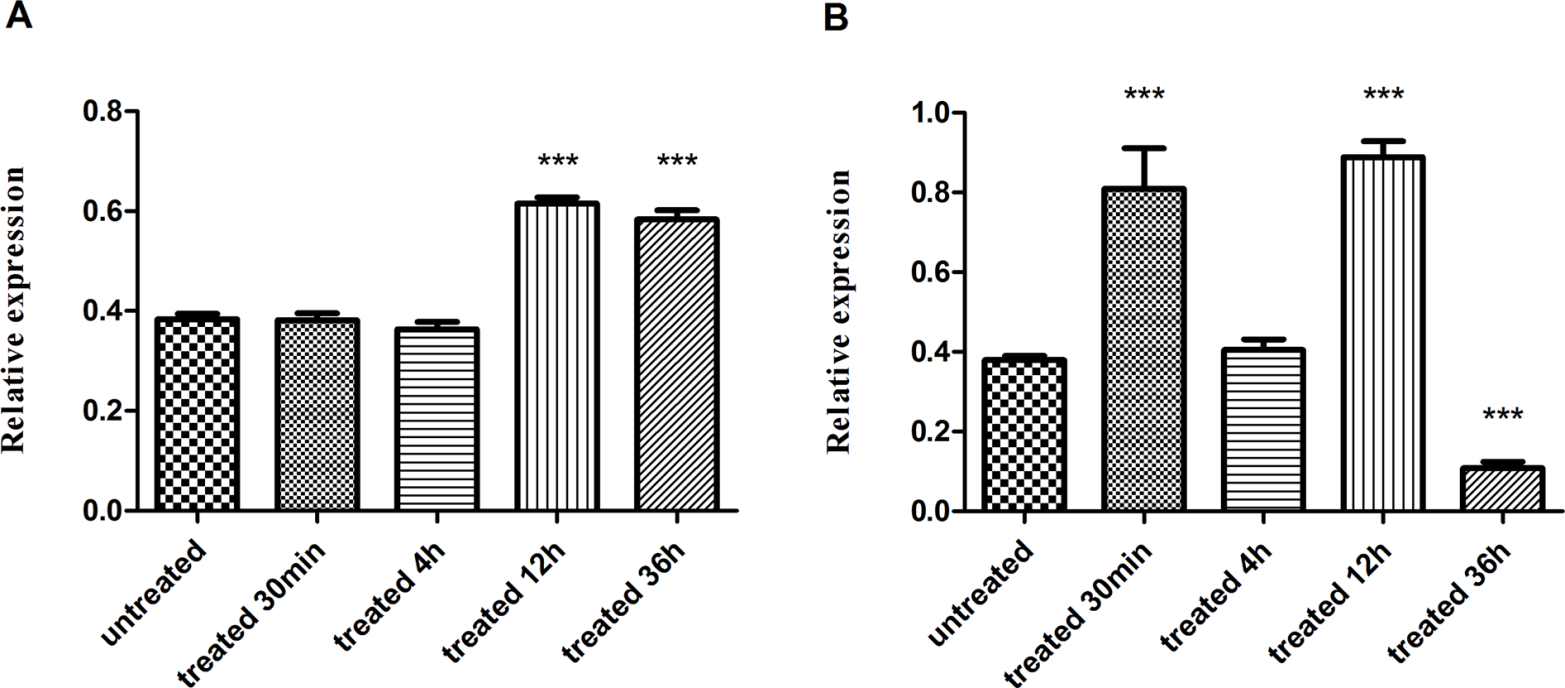
Effect of PZQ on SjVAMP2 transcription. (A) Mice were treated with a single dose of 40 mg/kg of PZQ (incomplete treatment dose) and sacrificed at various times post-treatment. (B) Mice were treated with single dose of 200 mg/kg of PZQ (treatment dose) and sacrificed at various times post-treatment. Statistically significant differences compared with the control group are denoted by *** (P<0.001).

After treated with 200 mg/kg PZQ for 36h, more than 60% worms died, and the remained worms curled, spastic paralyzed, twisted with stiffness, and the paired male and female worms separated. And the tegumental surface of some worms became ballooned and swollen. When treated with 40 mg/kg PZQ, the worms curled and twisted weakly at 4h after treatment, but most worms recovered with normal activities at 12h or 36h. SjVAMP2 transcript levels differed with different doses of PZQ treatment. Compared with the control, SjVAMP2 expression was significantly higher than the control at 12 and 36 h post-PZQ treatment with an incomplete treatment dose of 40 mg/kg of PZQ (p<0.001). However, this was not so for a treatment dose of 200 mg/kg of PZQ, in which case SjVAMP2 transcription increased to around twice that of the untreated control after 30 min or 12 h of treatment, and significantly decreased at 36 h (p<0.001) (Fig 4).

### Cloning and expression of SjVAMP2

An expected 483-bp cDNA sequence encoding the SjVAMP2 ORF was obtained by PCR using specific oligonucleotides (S3 Fig). After the PCR product was ligated into pET28a(+) vector and transformed into *E*. *coli* BL21(DE3) cells, the expression of pET28a(+)/SjVAMP2 was induced by IPTG, then purified by Ni-affinity chromatography. SDS-PAGE analysis revealed that rSjVAMP2 existed mainly as inclusion bodies with a band corresponding to 25 kDa, which was consistent with the predicted molecular mass of the His-tagged SjVAMP2 protein (S4 Fig).

### Immunogenicity Analysis of rSjVAMP2

Western blotting showed that the polyclonal anti-rSjVAMP2 serum specifically recognized SWAP and SEA, while no positive band was detected in the healthy mouse serum group (S5 Fig).

### Distribution of SjVAMP2 in *S*. *japonicum*

**Fig 5:**
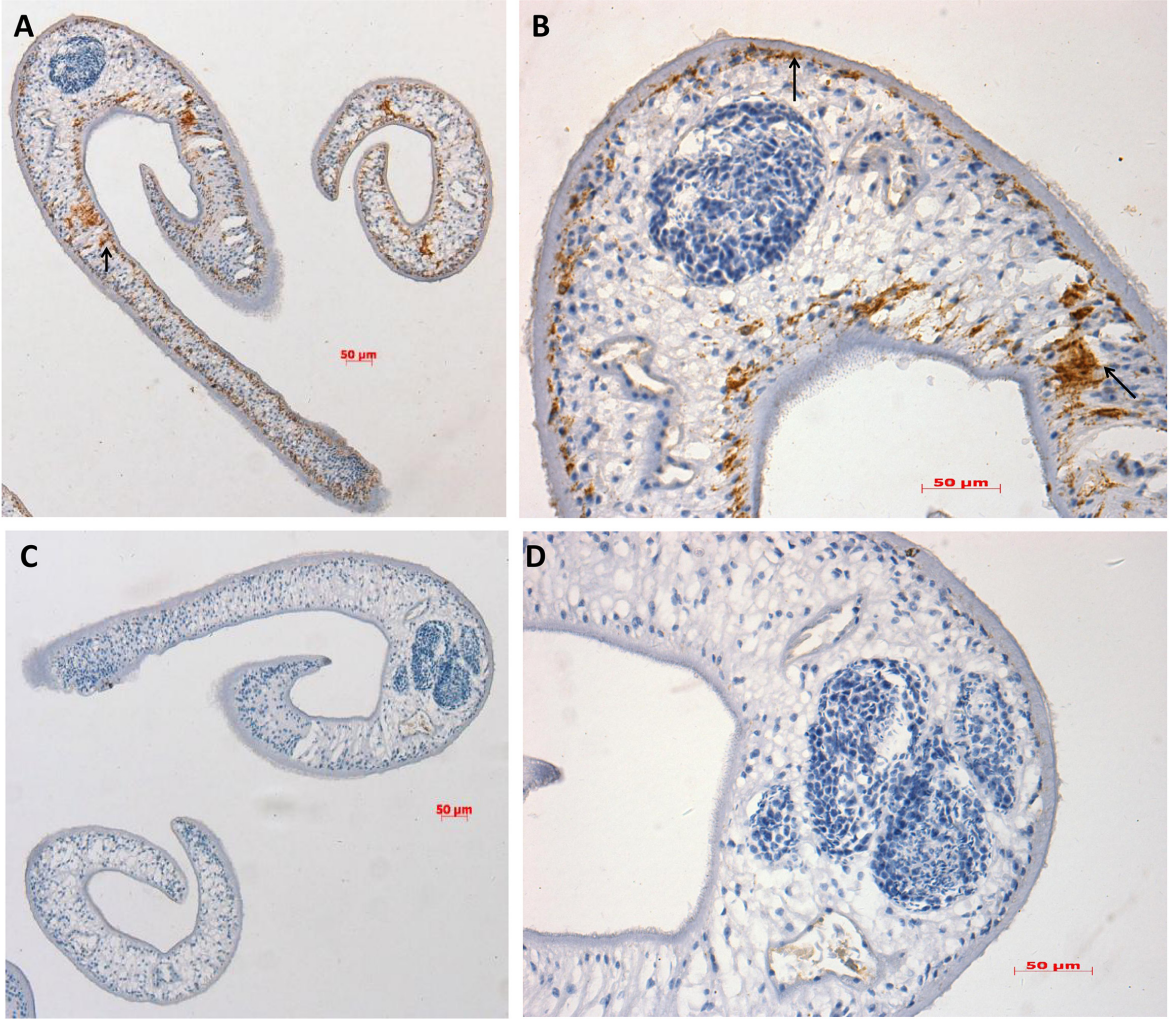
Immunolocalization of SjVAMP2 in *S*. *japonicum*. A, B, section of 28-day-old worm probed with anti-rSjVAMP2 mouse serum; C, D, section of 28-day-old worm probed with native mouse serum (negative control). The brown color indicates binding of the anti-rSjVAMP2 mouse serum, and the arrows show the location of the SjVAMP2 protein. The slides were analyzed by brightfield microscopy (A, C, magnification, ×100; B, D, magnification, ×400).

Immunostaining method was performed to identify the distribution of the SjVAMP2 protein in the 28d parasites with anti-rSjVAMP2 or naive mouse serum. The results showed that SjVAMP2 was mainly distributed in the sub-tegument and the internal tissues of the parasite (Fig 5A and 5B), while no specific staining was observed in sections incubated with serum from healthy mice (Fig 5C and 5D).

### Mouse vaccination experiment

All data in different tested groups passed the normality test. The number of adult worms recovered and hepatic eggs per gram in rSjVAMP2 vaccination group was significantly lower than that in the ISA 206 adjuvant control group at week 6 post-challenge as shown in Table 1. rSjVAMP2 vaccination lead to a 41.5% reduction (P<0.001) in number of worms and 36.8% hepatic egg burden reduction (P<0.05) compared to the ISA 206 adjuvant group. In a repeat vaccination trial, a 27.3% worm reduction (P<0.01) and 23.3% hepatic egg burden reduction (P<0.05) were obtained in the rSjVAMP2 vaccination group. At the same time, there were no significant differences between the ISA206 adjuvant group and the PBS group.

**Table 1:** Comparison of the number of adult worms and EPG between the rSjVAMP2-immunized group and the control groups.

	Group	Average number of worms (X±SD)	Average number of EPG (X±SD)
Trial 1	rSjVAMP2	11.25±1.38*** (41.5%)	20268±9267* (36.8%)
	ISA 206	19.25±1.67	32062±6694
	PBS	20.56±2.60	32387±144744
Trial 2	rSjVAMP2	19.75±4.53** (27.3%)	26351±6126* (23.3%)
	ISA 206	27.00±2.67	34348±6126
	PBS	25.10±2.81	40131±6894

Data are expressed as means ± SD and statistically significant differences compared with the ISA 206 adjuvant groups are shown by

* (P<0.05)

** (P<0.01) or

*** (P<0.001).

### Detection of rSjVAMP2-specific IgG antibodies

**Fig 6:**
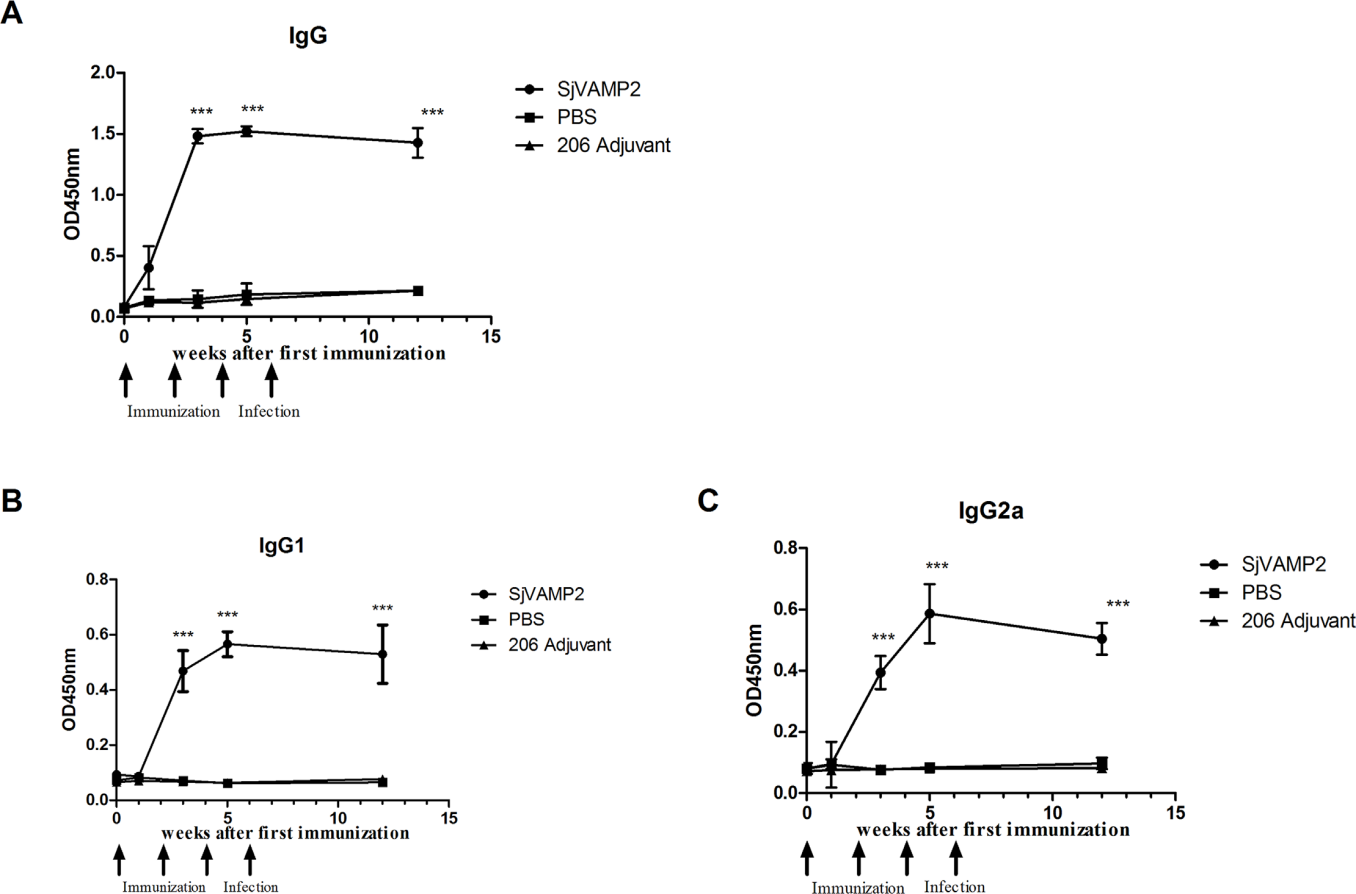
rSjVAMP2-specific IgG, IgG1 and IgG2a responses in different groups. Mice were immunized with rSjVAMP2, ISA206 adjuvant, or PBS on weeks 0, 2, and 4, and they were challenged with cercariae on week 6. Serum samples were obtained from ten individual mice from each group on weeks 0, 1, 3, 5, and 12 before sacrifice, and assayed by ELISA. Values depicted are means ± SEM. *** (P<0.001) for the rSjVAMP2-vaccinated group compared with the control groups.

The level of IgG antibody specific to rSjVAMP2 in the sera was determined by ELISA. Compared with the ISA206 adjuvant or PBS control groups, the specific IgG in the rSjVAMP2 immunization group increased dramatically after the second immunization, and it remained elevated after the third vaccination, and was maintained at a high level until the mice were sacrificed. The trends of specific IgG1 and IgG2 subtype antibodies were similar to that of the total IgG antibody (Fig 6). It remained elevated after the second vaccination.

## Discussion

Membrane fusion is a key step in the process of subcellular compartmentalization, cell growth, hormone secretion and neurotransmission, which is driven by protein machineries, such as SNARE [14,25,26]. SNAREs have been best characterized in nerve cells, where they mediate the docking and fusion of synaptic vesicles at the nerve terminal plasma membrane [27,28]. Additionally, VAMP2 was reported to contain an excess number of positively charged residues, which may interact with acidic lipids and modulate the fusion of transport vesicles with the plasma membrane [29,30]. Mariela *et al*. revealed that VAMP2 can mediate cAMP-stimulated renin release in mouse juxtaglomerular cells [31]. Even though the essential role of VAMP2 has been best recognized in many aspects, its impact on the development of *S*. *japonicum* is unknown.

SjVAMP2 appears to be a member of synaptobrevin superfamily in *S*. *japonicum*, and it might be involved in the targeting and/or fusion of transport vesicles to their target membrane via v-SNARE N-coiled-coil homology domain, as is VAMP2 in other species [32]. Additionally, SjVAMP2 has a conserved C-terminus domain that is not present in mammals, which implies that it may play a special role in worm development. Its biologic function needs to be explored further.

The tegument of schistosomes is a continuous unit, or syncytium of fused cells around the entire worm with a single continuous double-bilayer membrane. The underlying tegumental cytoplasmic layer or syncytial-matrix contains numerous discoid bodies and multilaminate vesicles, which appear to be derived from sub-tegument cells and transported to the tegument by cytoplasmic channels [7,9]. It is suggested that these discoid bodies and multilaminate vesicles have a function in formation and replacement of the outer-surface membrane by fusing to the tegument [33]. In accordance with the recent proteomics studies in our laboratory, immunostaining assay showed that SjVAMP2 was primarily located in the sub-tegument.

Besides, the RT–PCR result showed that SjVAMP2 was expressed at higher level in 14-, 28- and 42-day-old worms, and its expression was significantly higher in 42-day-old female worms than in male worms. The high level may be due to the fact that female worms begin or lay egg in a large amount during this developmental stage. *S*. *japonicum* egg production commences at 25 days after infection, peaks at the 42 day [2,4,34]. And more molecules like glucose required to be transported via vesicle trafficking to support egg production [35]. The transport protein SGTP4 is proved located at tegumental multilamellar bodies, discoid bodies, and the surface lipid bilayers [36]. And sperm-egg fusion is an intricate process, during which exocytosis of acrosome—the sperm’s single vesicle depends on activation of neurotoxin-sensitive SNAREs, and BoNT/B is capable of inhibiting the process by cutting VAMP [37–39]. Besides, there is a rapid increase in size of the worms and the surface does become increasingly folded and pitted with the worms grow and mature. Thus the large increase in surface area of the worm must require further formation of new outer membrane. Studies have shown that the tegument of adult worms contains significant numbers of membranous vesicles or multilamellar bodies [40–42]. And it is reported that the fusion of myoblasts during muscle cell development requires SNARE- and VAMP2-mediated, insulin-dependent incorporation of GLUT4 into the plasma membrane of L6 myoblasts [43]. This suggests that SjVAMP2 may be involved in membrane fusion and appears to be important for membrane formation/replacement or egg production of schistosomes. Further RNAi experiments may help us to elucidate the role of SjVAMP2 in the membrane formation process.

Our previous observation revealed that when schistosomes were treated with a complete dose (200 mg/kg) of PZQ, the tegument and sub-tegument of the worms were severely damaged, which finally resulted in the deaths of the treated worms. However, when the worms were treated with an incomplete dose (40 mg/kg) of PZQ, the damaged tegument recovered gradually post-treatment [44]. In this study, we noticed the similar result. And SjVAMP2 was up-regulated significantly at 12 h and 36 h post incomplete dose (40 mg/kg) PZQ treatment. However, when treated with a complete dose of 200 mg/kg of PZQ, which is deadly, the expression of SjVAMP2 increased significantly at 30 min and 12 h post-treatment, but declined thereafter. The different responses to high or low PZQ treatment mean that PZQ has an effect on SjVAMP2 expression. PZQ is the only choice for schistosomiasis treatment. Despite of numerous in-depth research, the mechanism of action of PZQ remains poorly defined. However, the disruption of ion transport [45], the alteration of schistosomal membrane fluidity, and the inhibition of nucleoside uptake were well considered being related to the mechanisms of PZQ action on schistosomes [46,47]. Besides, study have shown that when adult worms recovered from mice are maintained in vitro in monkey anti-mouse serum, an increased number of membraneous bodies can be detected. The worm probably responds by casting off the damaged membrane caused by antibodies and replacing it with new membrane from the membraneous bodies [41]. It is suggested that the membraneous bodies appear to be involved in the repair of damaged outer membrane. And Dumaine et al. have proved that electrostatic interactions between VAMP2 and acidic phospholipids may modulate the fusion of transport vesicles with the plasma membrane [29]. Therefore, we can speculate that SjVAMP2 may serve to maintain the function of the plasma membrane of *S*. *japonicum*, and it may be involved in the recovery of the tegument and other structure post PZQ treatment, although this needs to be confirmed. Ongoing studies, like electron microscopy observation of PZQ damage on tegument will help us understand the role of SjVAMP2 in tegument recovery, and the mechanism of PZQ as well.

Although comprehensive measures including community chemotherapy, snail control, and education and sanitation improving are important for reducing schistosomiasis prevalence and morbidity in endemic areas, reinfection and the low, but persistent levels of schistosomiasis transmission highlights the importance of exploring new potential vaccine molecules for the control of this disease [48,49]. Proteins on the surface of the tegument of schistosomes are always considered to be essentially important molecules, as they are directly exposed to the host immune system. A comprehensive analysis of host immune responses to schistosome infection at a proteome-wide level is an essential step toward the identification of novel antigen targets for vaccines [11,12]. In a recent proteomic study of *S*. *japonicum* in our laboratory, we identified SjVAMP2 and 84 other proteins from the tegument of 42-day-old adult worms, which provided the possibility of identifying new, effective molecules for vaccine candidates. Western blotting showed that rSjVAMP2 had good immunogenicity. In our two independent immunization tests in this study, rSjVAMP2 vaccination elicited significant worm burden reductions of 41.5% and 27.3%, and hepatic egg numbers reductions of 36.8% and 23.3% compared with ISA206 adjuvant groups. The results showed that rSjVAMP2 vaccination could induce partial, but significant, protection against schistosome infection in mice, which is consistent with previously published studies of the tegument proteins Sm29 or SmTSP [50,51].

Because schistosomes have a complicated life cycle with multiple stages of development during migration in different tissues of its host, a single protein vaccine may not be sufficient to protect the host against parasite invasion. Additionally, the well-known immune evasion and rapid regenerating tegument of worms make it more difficult to develop an effective vaccine. Consequently, a breakthrough must be made to exploit new, effective vaccines, such as polyvalent vaccines [52–54]. And rSjVAMP2 may be an effective constituent of a polyvalent vaccine.

Throughout the vaccination-challenge trial period, the rSjVAMP2 immunization group developed stronger and more prolonged humoral immune responses in comparison with the control groups. As the data showed, rSjVAMP2 vaccinations elicited stronger specific IgG responses including IgG1 and IgG2a after the second immunization, and the high IgG level lasted. This specific IgG response might contribute to the anti-infection protection induced by rSjVAMP2. The results suggest that rSjVAMP2 appears to be a good vaccine candidate.

## Conclusion

In the study, we first cloned, expressed, and characterized the SjVAMP2 gene of *S*. *japonicum*. Vaccination with rSjVAMP2 could induce partial, but significant protection in mice. This study will serve as a fundamental basis for further insights into the biological function of SjVAMP2 in *S*. *japonicum*.

## Supporting Information

S1 Fig Transmembrane helix prediction of SjVAMP2. Transmembrane helix was predicted by online program of Phyre2 in the sequence of SjVAMP2 to adopt the topology. The transmembrane domain includes 87 to 109 amino acid residue.
(TIF)

S2 Fig Putative three dimensional structure of SjVAMP2. The tertiary structure of SjVAMP2 was predicted by online program of Phyre2. N-coiled coil domain is the N-terminal v-SNARE coiled-coil homology domain. The secondary structure of SjVAMP2 was predicted to have ɑ-helix.
(TIF)

S3 Fig Agarose gel electrophoresis of PCR products for SjVAMP2. M, 2000-bp DNA ladder; Lanes 1, 2, and 3, PCR products of SjVAMP2.
(TIF)

S4 Fig SDS–PAGE analysis of purified rSjVAMP2. M, protein molecular marker. lane 1, purified rSjVAMP2 protein.
(TIF)

S5 Fig Western blotting analysis. Lane M: molecular mass marker; lane 1: souble 42d-worm antigen probed with serum from mice immunized with rSjVAMP2.
(TIF)
