# Supplementary figures and images for: Characterization of VAMP2 in Schistosoma japonicum and the Evaluation of Protective Efficacy Induced by Recombinant SjVAMP2 in Mice
Source: PLoS One. 2015 Dec 7;10(12):e0144584. doi: 10.1371/journal.pone.0144584 (PMC4671580; doi:10.1371/journal.pone.0144584)

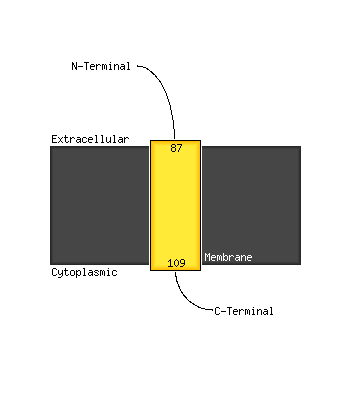

Supplement: S1 Fig — Transmembrane helix was predicted by online program of Phyre2 in the sequence of SjVAMP2 to adopt the topology. The transmembrane domain includes 87 to 109 amino acid residue. (TIF) [file pone.0144584.s001.tif]

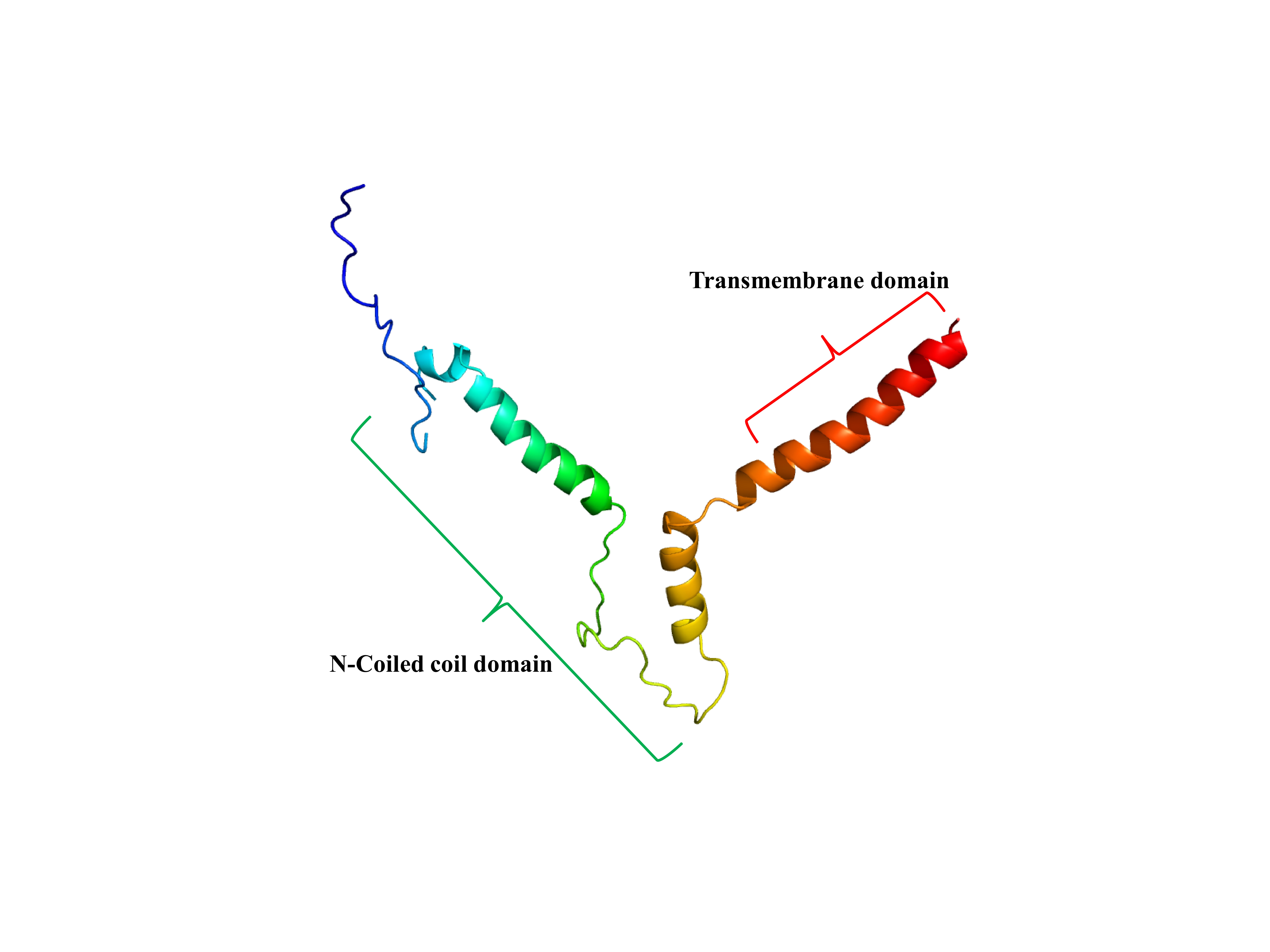

Supplement: S2 Fig — The tertiary structure of SjVAMP2 was predicted by online program of Phyre2. N-coiled coil domain is the N-terminal v-SNARE coiled-coil homology domain. The secondary structure of SjVAMP2 was predicted to have ɑ-helix. (TIF) [file pone.0144584.s002.tif]

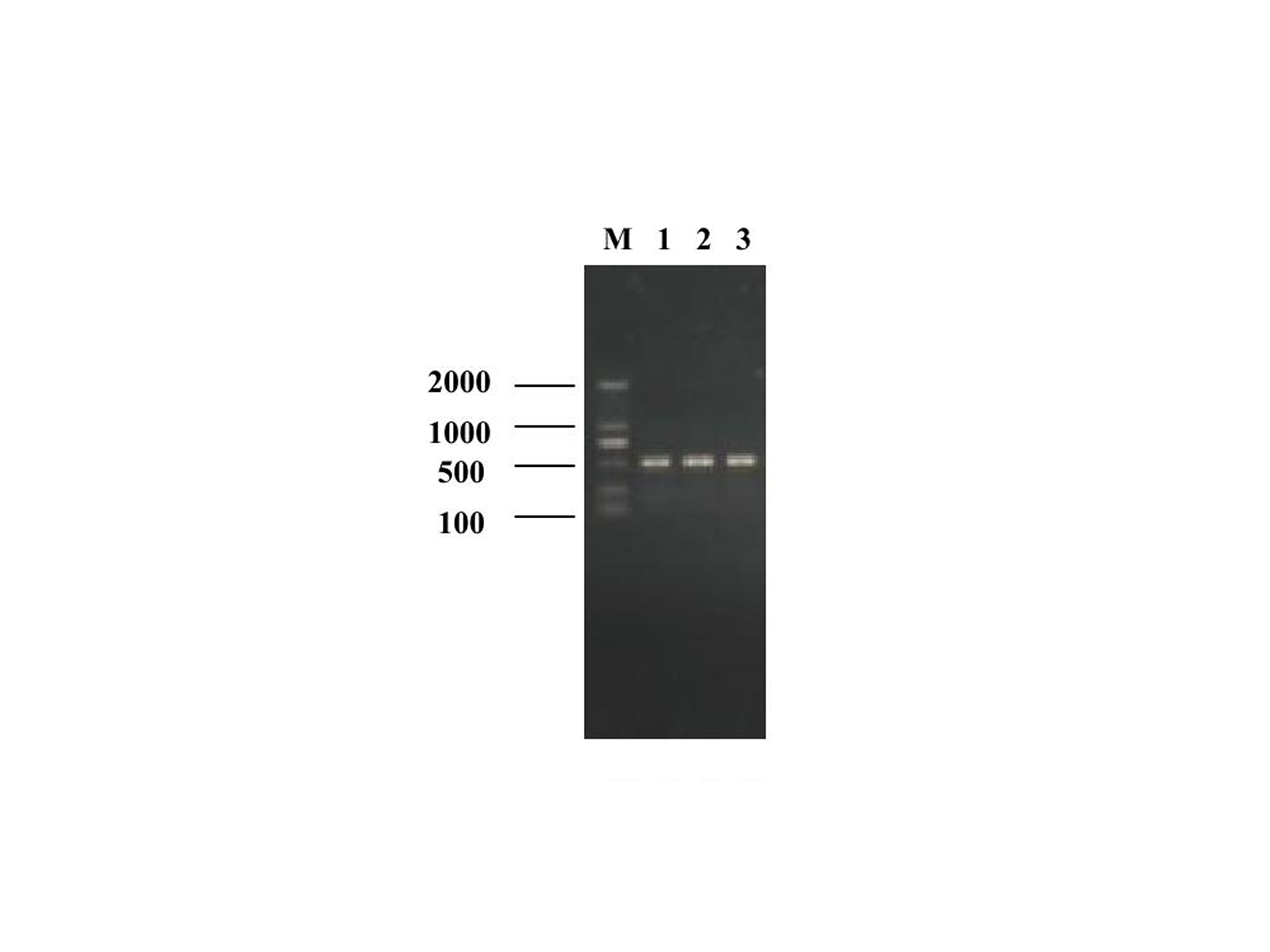

Supplement: S3 Fig — M, 2000-bp DNA ladder; Lanes 1, 2, and 3, PCR products of SjVAMP2. (TIF) [file pone.0144584.s003.tif]

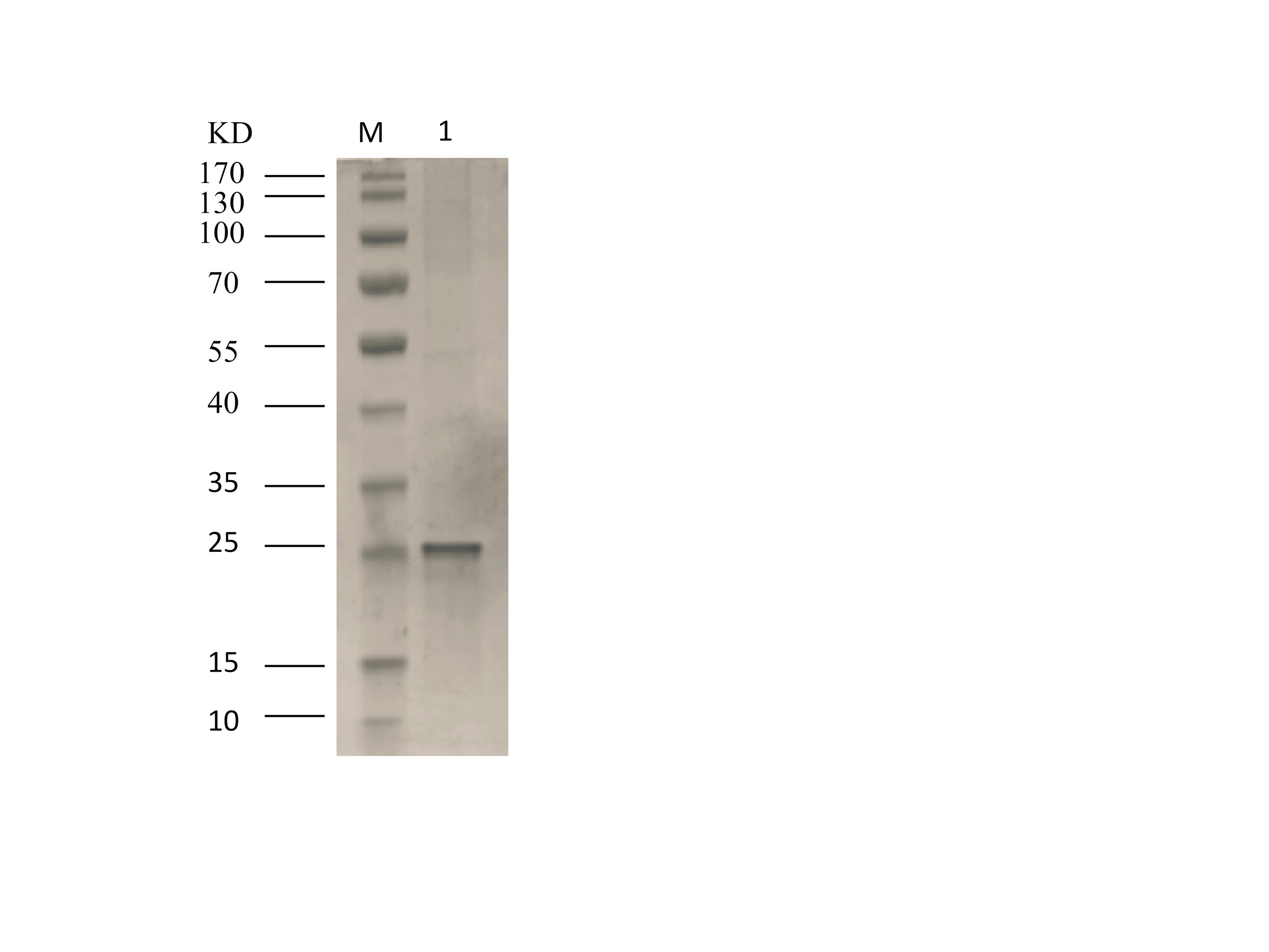

Supplement: S4 Fig — M, protein molecular marker. lane 1, purified rSjVAMP2 protein. (TIF) [file pone.0144584.s004.tif]

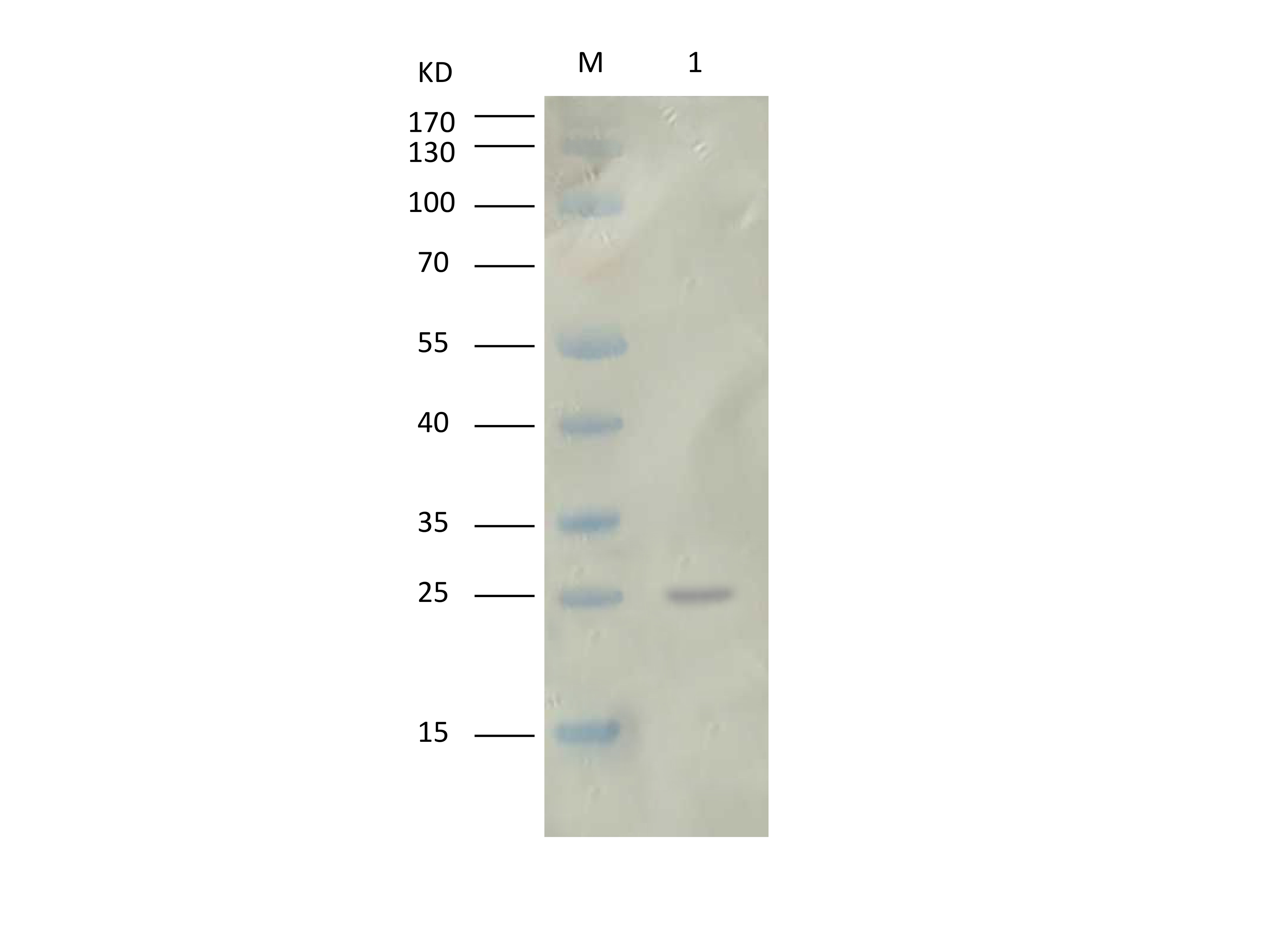

Supplement: S5 Fig — Lane M: molecular mass marker; lane 1: souble 42d-worm antigen probed with serum from mice immunized with rSjVAMP2. (TIF) [file pone.0144584.s005.tif]
